# Enhancing the SCC Resistance of the Anchor Steel with Microalloying in a Simulated Mine Environment

**DOI:** 10.3390/ma16175965

**Published:** 2023-08-31

**Authors:** Hailong Du, Na An, Xiyan Wang, Yongliang Li, Zhiyong Liu, Aibing Jin, Renshu Yang, Yue Pan, Xiaogang Li

**Affiliations:** 1 School of Civil and Resource Engineering, University of Science and Technology Beijing, Beijing 100083, China; 13703561606@163.com (H.D.);; 2 Technology Research Institute of Shanxi Jincheng Coal Group Co., Ltd., Jincheng 048000, China; 3 National Materials Corrosion and Protection Scientific Data Center, Key Laboratory for Corrosion and Protection (MOE), University of Science and Technology Beijing, Beijing 100083, Chinab20150517@xs.ustb.edu.cn (X.W.);; 4 School of Energy and Mining Engineering, China University of Mining and Technology-Beijing, Beijing 100083, China; 5 Hebei Special Equipment Supervision and Inspection Institute, Key Laboratory of Safety Evaluation of Steel Pipes and Fittings for State Market Regulation, Shijiazhuang 050061, China

**Keywords:** microalloying, mining anchor bolts, SCC, hydrogen embrittlement

## Abstract

This work explored a new idea for enhancing the resistance to stress corrosion cracking (SCC) of mining anchor steel through microalloying. Microalloyed anchor steels with Nb, Cu, Ni, Sb, and C were prepared through vacuum smelting and hot rolling. Electrochemical measurements, slow strain rate tensile (SSRT) tests, and fracture morphology observations were used to study the electrochemical and SCC behavior in the simulated mine environment. The results proved that the microstructure of microalloyed steels varies slightly. Adding Ni, Cu, and Sb can improve the mechanical properties of the anchor steel, while reducing C content decreases tensile strength as a result of loss of the solution-strengthening effect. The addition of Sb, Cu, Ni, and reducing the content of C enhances the resistance to corrosion and SCC by mitigating anodic dissolution (AD), while adding Nb improves SCC resistance by inhibiting hydrogen embrittlement (HE). The combined addition of 1% Ni, 0.5% Cu, 0.05% Nb, 0.1% Sb, and 0.5% C presented the highest SCC resistance, which is a promising prospect for the development of high-performance, low-alloy anchor steels. The combined addition of 1% Ni, 0.5% Cu, 0.05% Nb, and 0.1% Sb resulted in the inhibition of electrochemical reactions and corrosion. As a result of the synergistic effect of the microalloy, both AD and HE mechanisms were simultaneously inhibited, which greatly enhanced SCC resistance.

## 1. Introduction

Anchoring technology has been widely used in all types of mines, but the service conditions of the anchor cables and rods have been deteriorating year by year due to the increasing mining depth and corrosion problems in various aggressive mine environments. According to reported statistics [1,2], 29% of anchor bolts and 25% of anchor cables in the Australian mining industries are subjected to corrosion failures, and corrosion has been recognized as one of the biggest threats to the life of anchor bolt structures buried in rock and soil layers. In addition, at external load or residual stress, stress corrosion cracking (SCC) and hydrogen embrittlement (HE) happen frequently and lead to abrupt failures [3,4,5]. Therefore, SCC and HE problems pose a serious threat to the safe service of anchor bolt structures and severely affect the development and application of anchor steels.

Studies have shown that high-strength anchor steels are vulnerable to SCC in chloride-containing environments [6,7]. The SCC mechanism is considered a mixture of anodic dissolution (AD) and hydrogen embrittlement (HE). Microalloying has become the mainstream method for optimizing corrosion and SCC resistance by inhibiting AD and HE. It has been reported that Ni, Nb, Cu, Sb, and C have a great impact on corrosion and SCC. In general, Ni, Cu, and Sb can mitigate AD, while adding Nb prevents HE. It has been reported that Ni can greatly enhance the corrosion resistance of steels by improving the compactness of the rust layer [8,9]. Adding Cu can improve corrosion resistance because it facilitates CuO formation and enhances the protective capacity of the corrosion product layer [8]. In addition, in Ni-bearing steels, a small amount of Cu addition can be beneficial to the function of Ni in improving the rust layer protectiveness [10]. Sb is an acid-resistant element and has a synergetic effect with Cu and Ni, which can inhibit AD and improve corrosion resistance [11]. Previous work has proved that Sb induces the enrichment of Cu and Ni and improves the resistance of the rust layer to corrosive chloride ions [11]. Therefore, the combined addition of Ni, Cu, and Sb should be effective at improving the SCC resistance of anchor steels by mitigating AD. Nb can mitigate hydrogen-induced cracking problems by inhibiting crack initiation and propagation [12] because Nb addition can facilitate the formation of nanoprecipitated Nb(C, N), which can act as hydrogen traps and prevent hydrogen diffusion [13]. Adding C can refine grains and improve the strength of steels by the solution-strengthening mechanism but has an opposite effect on corrosion behavior. C addition can facilitate carbide formation at grain boundaries and cause intergranular corrosion [14]. Hence, the addition of Nb and adjusting the content of C are also expected to enhance the resistance to corrosion and SCC. The above research has demonstrated that microalloying is feasible in mitigating the SCC of low-alloy steels. However, few studies have paid attention to the microalloy effect on the SCC behavior and mechanism of anchor steels.

Therefore, based on the above progress, in this work, a series of anchor cable steels were prepared by adding Nb, Sb, Cu, Ni, and reducing C content. The effect of microalloying on the SCC behavior and mechanism was analyzed systematically, which is anticipated to propose new ideas and theoretical support for the application and safe service of anchor steels.

## 2. Experimental Procedures

### 2.1. Materials and Solution

**Figure 1:**
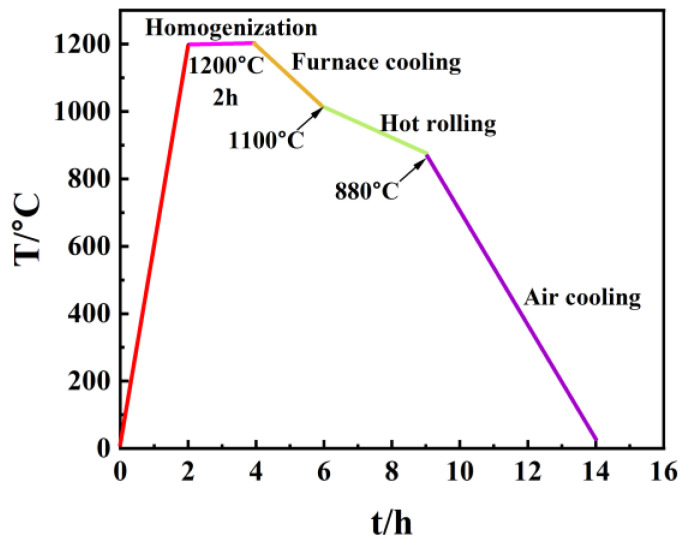
Schematic diagram of the heat treatment process for the prepared low-alloy anchor steels.

Four types of low-alloy, ultra-high-strength anchor steels with different chemical compositions were prepared, and the chemical composition is listed in Table 1. Commercial YL82B steel was selected as the as-received material, which is labeled as the BM in this work. Three other kinds of steels were added to alloy elements compared to the BM. 0.5NiCu denotes the addition of 0.5% Ni with 0.5% Cu and other trace alloying elements (0.046% Nb + 0.11% Sb). 1NiCu represents the adjustment of Ni elements to 1% and the decrease in carbon content to 0.53% compared to 0.5NiCu. LNiCu represents the decrease in carbon content to 0.34% in comparison to 1NiCu as the decarburized version of 1NiCu. The ingots were prepared by vacuum smelting in a vacuum induction furnace, and the procedures are illustrated in Figure 1. As shown, the ingots were homogenized at 1200 °C for 2 h, furnace-cooled at 1010 °C, and then hot-rolled at the final temperature of 880 °C followed by air cooling. All the test specimens in this work were taken from the center thickness of the steel sheet to avoid the possible errors caused by surface decarburization during hot rolling.

The corrosive medium in this work was the simulated mine environment, and the chemical composition is shown in Table 2 according to the analysis of the chemical composition of the groundwater and soluble gas contents in underground coal mines. The solution pH was adjusted to 5.0 with acetic acid. All the ingredients were analytically pure for the preparation of the simulated solution, and all the tests were performed at ambient temperature.

### 2.2. Microstructural Analysis

Metallographic samples with size of 10 mm × 10 mm × 3 mm were cut from the rolling and transverse (RD–TD) planes. Then, all samples were ground to 3000 grits with SiC paper, mechanically polished to 1 μm, and chemically etched with 4% nital solution. The microstructure was observed with an FEI Quanta 250 scanning electron microscope (Eindhoven, The Netherlands) (SEM).

### 2.3. Mechanical Property Test

**Figure 2:**
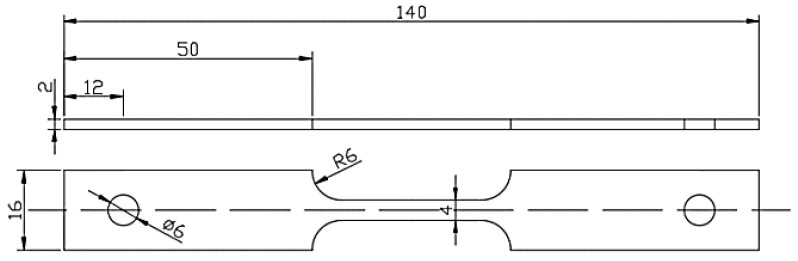
Schematic diagram of tensile specimens.

Tensile tests were carried out to study the mechanical properties of the test steels. The test specimens were cut from the rolling–transverse (TD–RD) plane along the rolling direction, and the dimensions are shown in Figure 2 in accordance with the Chinese National Standard GB/T228.1-2021 [15]. Prior to the test, the gauge length of the specimens was sequentially ground with SiC paper to 3000#, and the final grinding direction was parallel to the gauge length. The tensile tests were performed with an MTS-Landmark-730 hydraulic servo fatigue testing machine (Eden Prairie, MN, USA) with the tensile rate of 10^−3^ s^−1^. After fracture, the elongation and cross-sectional area were measured with a vernier caliper. All the tests were performed at least three times to ensure the repeatability and reliability of the results.

### 2.4. Electrochemical Measurements

The electrochemical properties of the test steels were measured with a VersaSTAT3 electrochemical workstation produced by Princeton Applied Research (Trenton, NJ, USA). The three-electrode system was applied, in which the saturated calomel electrode (SCE) was used as the reference electrode, a platinum plate as the counter electrode, and the test steel with the dimension of 10 mm × 10 mm × 3 mm as the working electrode. The test specimens were sealed with epoxy resin except for the 1 cm^2^ working area, and the working surface was ground with SiC paper sequentially to 3000 grits and washed with deionized water and alcohol, followed by blow drying. Prior to the test, the air-formed oxide film was removed through potentiostatic polarization at −1.2 V (vs. SCE) for 60 s, and then the open-circuit potential (OCP) was recorded for at least 30 min until reaching a steady state. The electrochemical impedance spectroscopy (EIS) measurements were performed at the AC excitation amplitude of 10 mV and the test frequency ranging from 100 kHz to 10 mHz. Potentiodynamic polarization measurements were conducted at the scan range of −1.2 V to −0.4 V (vs. SCE), and two scan rates were chosen: 0.5 mV/s to study the quasi-steady electrochemical process at the crack wall and 200 mV/s to study the non-steady electrochemical process at the crack tip [16]. All experiments were repeated more than three times in order to ensure repeatability and accuracy.

### 2.5. SSRT Tests

Slow strain rate tensile (SSRT) tests were performed to study the SCC susceptibility of the test steels. The dimension and the preparation of test specimens were the same as procedures described in Section 2.2. The SSRT tests were carried out at a strain rate of 10^−6^ s^−1^ with a WDML-30 kN material testing system. Each type of test steel was tested in air, under OCP and −1200 mV (vs. SCE) to study the SCC in the test solution without/with hydrogen charging. Prior to the test, specimens were preloaded with approximately 500 N for 12 h in the test solution in order to make sure that a uniform electrolyte film formed. For specimens tested at −1200 mV, potentiostatic polarization was imposed before and throughout the test. To ensure repeatability, all tests were performed at least three times. The elongation loss (*I_σ_*) and the loss of the reduction in area (*I_Ψ_*) were calculated with Equations (1) and (2) for characterizing the SCC susceptibility.

(1) $$I_{\sigma }=\left(1-\frac{\delta_{s}}{\delta_{0}}\right)\times 100\%$$

(2) $$I_{\psi }=\left(1-\frac{\psi_{s}}{\psi_{0}}\right)\times 100\%$$

where *δ_s_* and *δ*_0_ are the elongation rates of specimens tested in solution and air, respectively; *Ψ_s_* and *Ψ*_0_ are the reductions in area of the specimens tested in solution and air, respectively. However, the strengths of the four kinds of test steels are different, which makes the above equations fail to characterize the real SCC susceptibility differences among the different test steels. For example, under the same external stress, when the steel with a lower yield strength passes the yield point, the steel with a higher yield strength is below the yield point. This causes the steel with the higher yield strength to present lower *I_σ_* and *I_Ψ_* values than that with the lower yield strength. Therefore, we used the normalized elongation loss (*I_σx_’*) and the normalized loss of the reduction in area (*I_Ψx_’*) to evaluate SCC susceptibility, and the formulas for calculating them are as shown in (3) and (4):

(3) $${I_{\sigma x}}^{\prime }=\frac{I_{\sigma }}{\left(\sigma_{sx}/\sigma_{s0}\right)}\;(x=1,2,3)$$

(4) $${I_{\Psi x}}^{\prime }=\frac{I_{\Psi }}{\left(\sigma_{sx}/\sigma_{s0}\right)}\;(x=1,2,3)$$

where *σ_sx_* (*x* = 1, 2, 3) is the yield strength of the 0.5NiCu, 1NiCu, and LNiCu anchor steel; *σ_s_*_0_ is the yield strength of the BM.

After the SSRT tests, the fracture surface was cut for the morphology observation, and then the corrosion products were removed by ultrasonic cleaning in the mixed solution of 500 mL HCl + 500 mL H_2_O + 5 g hexamethylenetetramine for about 4~8 s. The fracture surface morphology and secondary cracks were observed with an FEI Quanta 250 SEM.

## 3. Results

### 3.1. Microstructural Characterization

**Figure 3:**
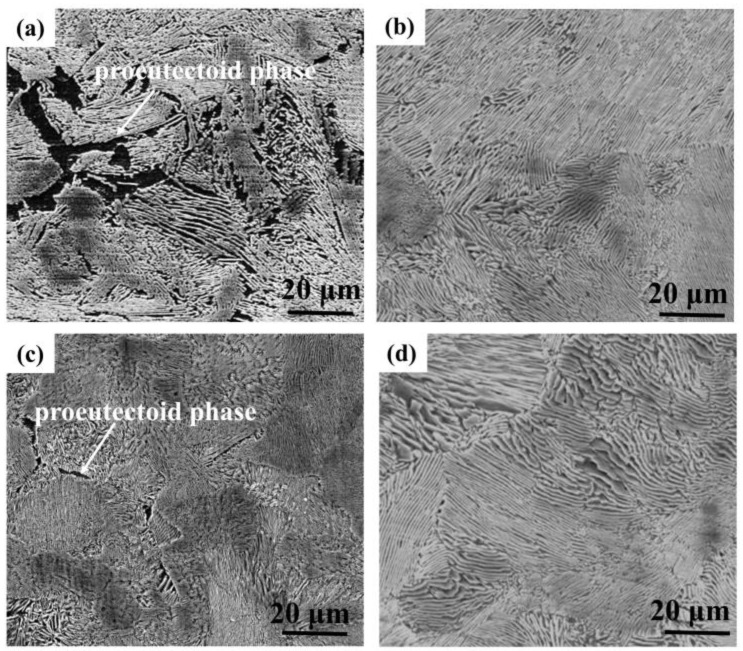
Microstructures of the prepared low-alloy, high-strength anchor steels: (**a**) BM; (**b**) 0.5NiCu; (**c**) 1NiCu; (**d**) LNiCu.

The microstructure of the test steels is shown in Figure 3. The microstructure of the four kinds of anchor bolt steels is mainly composed of pearlite, which appears as a fine lamellar structure. A small amount proeutectoid phase also exists and is inhomogeneously distributed as a result of the proeutectoid phase transformation, which is common in hypoeutectoid steels [17,18]. The results indicated that microalloying and decarburization (as represented by LNiCu) had no obvious influence on the steel microstructure.

### 3.2. Mechanical Properties

**Figure 4:**
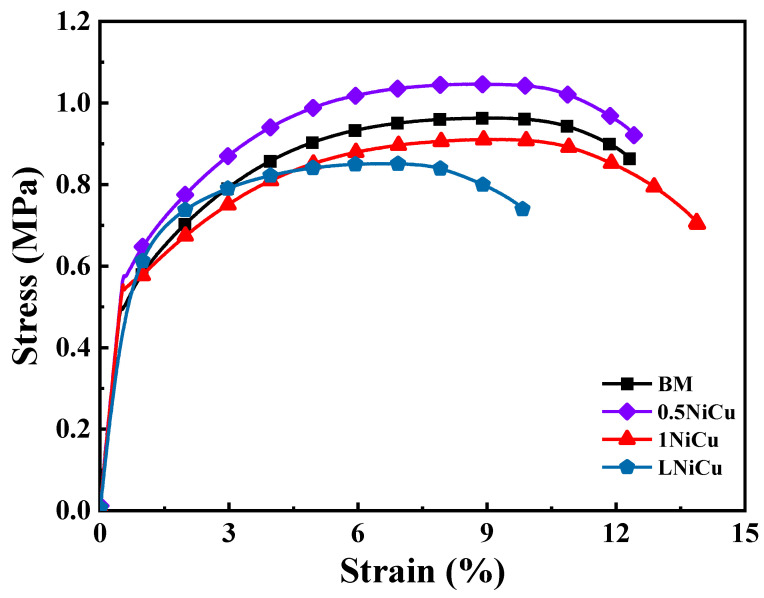
Stress–strain curves of the four kinds of anchor steels.

Stress–strain curves for the four test steels are shown in Figure 4, and the mechanical properties are listed in Table 3. The tensile strength of 0.5NiCu is the highest (1046 MPa), while the elongation decreases slightly compared to the BM. Due to the addition of the alloying elements Cu, Nb, Ni, and Sb, the microalloyed anchor steels present a higher yield ratio. The tensile strength of the 1NiCu and LNiCu is slightly lower than that of the BM, and the tensile strength of the LNiCu is the lowest due to the loss of the solution-strengthening effect as a result of the decreased C content [19]. Therefore, decarburization causes a decrease in the tensile strength. The mechanical properties of the various designed microalloyed steels satisfy the basic requirements compared to the BM.

### 3.3. Electrochemical Properties

#### 3.3.1. EIS Analysis

**Figure 5:**
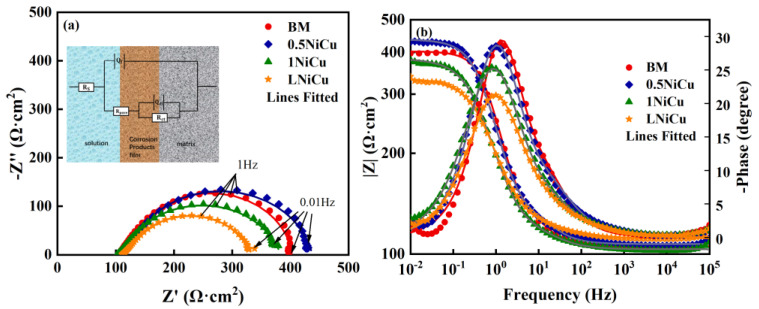
(**a**) Nyquist and (**b**) Bode plots of the prepared microalloyed anchor steels in a coal mine environment.

The EIS test results of the four test steels in the simulated mine environment are shown in Figure 5. All the Nyquist plots present a compressed semicircle, indicating a similar electrochemical mechanism. The semicircle radius of the 1NiCu steel is the biggest, which indicates that 1NiCu exerts the highest corrosion resistance. The Bode diagram indicates the existence of two time constants, and the Bode phase angle in Figure 5b is approximately 30° and far less than 90° on account of the porous corrosion product layer. Therefore, the equivalent electric circuit in the inset in Figure 5a with two time constants is used to fit the EIS curves, where *R_s_* represents the solution resistance, and *R_ct_* and *R_pore_* are the charge transfer resistance and corrosion product film resistance. *Q_dl_* and *Q_f_* are the constant-phase elements (CPEs) of the electric double layer and the corrosion product film, and the corresponding *n* values describe the deviation from an ideal capacitor caused by the inhomogeneity of the electrodes [20,21].

**Figure 6:**
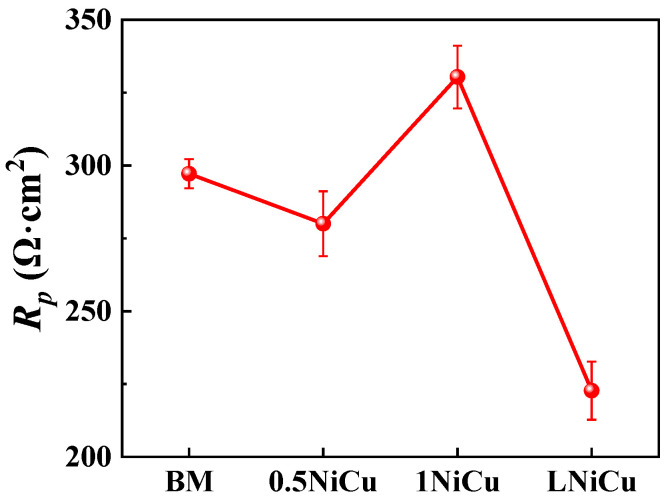
Statistical results of polarization resistance of the four kinds of anchor steels.

The fitted EIS values are listed in Table 4. The χ^2^ values are all at the 10^−4^ level, which indicates a high fitting quality. The values of *R_ct_* increase as a result of microalloying. In comparison to 1NiCu, *R_ct_* of LNiCu decreases and is lower than the BM. Therefore, it can be deduced that microalloying can suppress the charge transfer process, while decarburization accelerates charge transfer. The polarization resistance *R_p_* (*R_ct_ + R_f_*) is used to characterize the corrosion resistance of the test steels, and the values are shown in Figure 6. It has been widely acknowledged that *R_p_* is inversely proportional to the corrosion rate, so higher *R_p_* values indicate a higher corrosion resistance [22]. By comparing *R_p_*, we can conclude that 1NiCu presents the highest *R_p_* value and corrosion resistance, and LNiCu exerts the lowest *R_p_* and the worst corrosion resistance. In addition, 0.5NiCu exhibits a lower corrosion resistance than the BM, for Cu accelerates the generation of corrosion products and the corrosion process in the initial corrosion stage [23,24]. As a result, the corrosion resistance of Cr-bearing steels may decline. In addition, the beneficial effect of Cu on corrosion resistance makes a difference only when the content exceeds a certain value, so the corrosion resistance for 0.5NiCu is not markedly improved. The reason for the corrosion resistance differences is discussed thoroughly in the Discussion.

#### 3.3.2. Polarization Curve Analysis

**Figure 7:**
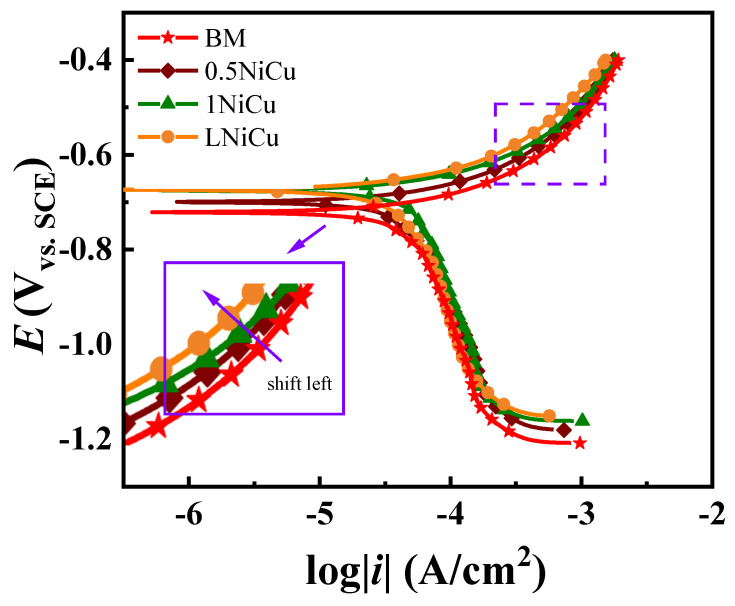
Polarization curves of the prepared microalloyed anchor steels in a coal mine environment.

Potentiodynamic polarization curves for the anchor steels at the 0.5 mV/s scan rate are shown in Figure 7. All the curves present a similar shape, which is an indication of a similar corrosion mechanism. The anodic curves present the active dissolution reaction, and the cathodic branches exert a combination of the oxygen reduction reaction and hydrogen evolution reaction. The inset figures show that the anodic curve shifts to the left with the effect of alloy elements and decarburization in comparison to the BM, which indicates that both the microalloy and reducing the carbon content inhibit anodic dissolution. As for the cathodic branch, the cathodic polarization curves of 0.5NiCu and 1NiCu shift to the right, while the curve of LNiCu almost overlaps with that of the BM. Considering the opposite variation tendency of the cathodic branch and the anodic branch, we did not conduct Tafel fitting for corrosion rates as referred to in the literature [25]. The polarization curves of the three kinds of microalloyed steels vary slightly.

### 3.4. SSRT Results

#### 3.4.1. Stress–Strain Curves and SCC Susceptibility

**Figure 8:**
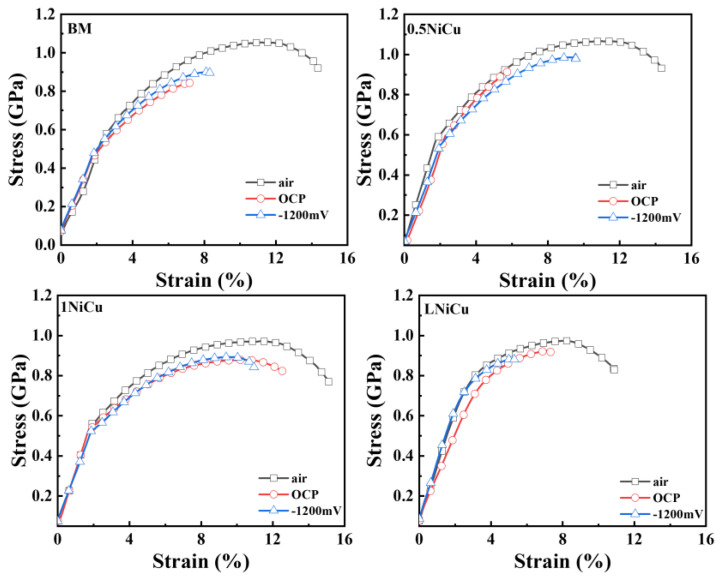
Stress–strain curves of the anchor steels after SSRT tests in air and simulated mine environment.

Figure 8 shows the SSRT stress–strain curves of the test anchor steels. In addition, the elongation loss of 1NiCu is the lowest in the test solution, indicating the highest SCC resistance. It is worth noting that the elongation of 0.5NiCu at −1200 mV is significantly higher than that at OCP, which is probably due to the hydrogen-induced plasticity effect. This phenomenon has been observed in previous work, which proves that hydrogen can release stress concentration at crack initiation sites, decreases the stress intensity, and postpones SCC initiation at the applied cathodic polarization [26,27].

**Figure 9:**
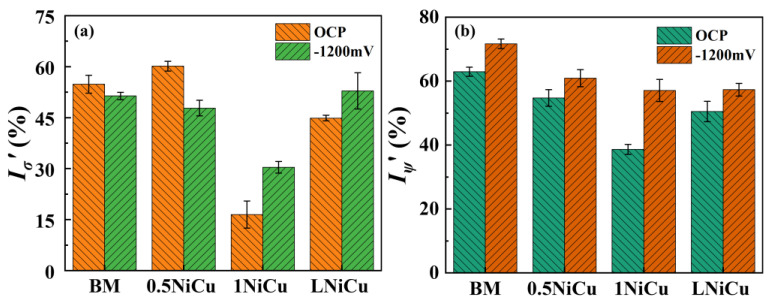
Normalized SCC susceptibility of the test anchor steels (**a**) ${I_{\sigma x}}^{\prime }$ and (**b**) ${I_{\Psi x}}^{\prime }$.

The normalized SCC susceptibility results of the test anchor steels are shown in Figure 9. When tested at −1200 mV, all microalloyed steels are less SCC-susceptible compared to the BM. Additionally, 1NiCu presents the highest SCC resistance at OCP and −1200 mV. Compared to the BM, the *I_σ_*’ of 1NiCu reduces by over 70% when tested at OCP, and reduces by 45% when tested at −1200 mV. LNiCu presents higher SCC susceptibility than 1NiCu. The reason is that the excessively low carbon content is harmful to the stability of austenite [28], which also causes microstructure degradation during phase transformation and deformation, leading to lower SCC susceptibility.

#### 3.4.2. Fracture Analysis

**Figure 10:**
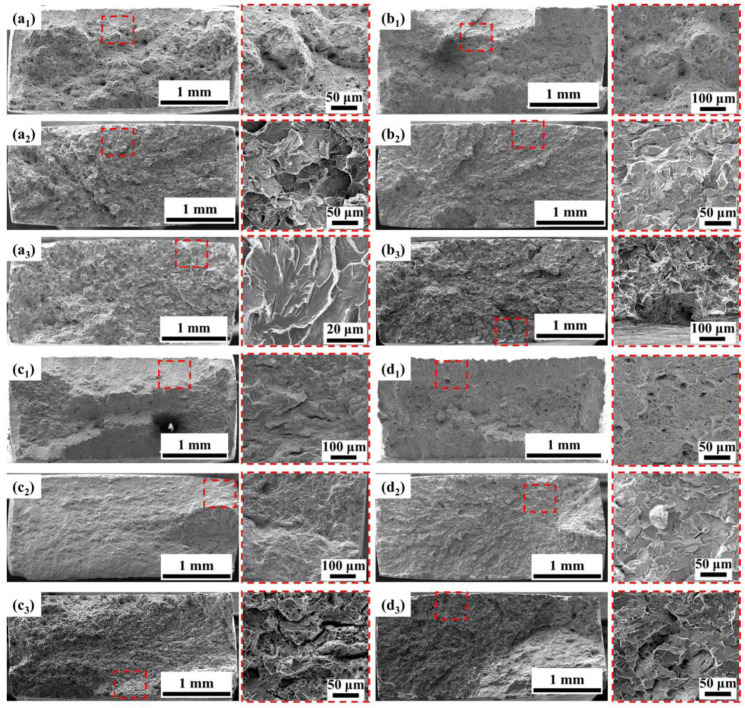
Fracture morphology of different test steels after SSRT tests in various test conditions: (**a_1_**) BM in air; (**a_2_**) BM at OCP; (**a_3_**) BM at −1200 mV; (**b_1_**) 0.5NiCu in air; (**b_2_**) 0.5NiCu at OCP; (**b_3_**) 0.5NiCu at −1200 mV; (**c_1_**) 1NiCu in air; (**c_2_**) 1NiCu at OCP; (**c_3_**) 1NiCu at −1200 mV; (**d_1_**) LNiCu in air; (**d_2_**) LNiCu at OCP; (**d_3_**) LNiCu at −1200 mV (The red squares show the crack initiation sites and the typical morphology).

After the SSRT tests, the fracture morphology of the anchor steels was carefully observed as shown in Figure 10. When the BM is tested in the air, there is obvious necking. In addition, dimples of different sizes are observed in the magnified view in Figure 10(a_1_), which are typical ductile fracture features. When tested in solution at OCP, the size and number of dimples decrease sharply, and brittle characteristics appear, indicating high SCC susceptibility. At −1200 mV, the brittle characteristics become more obvious. The SCC initiation site at the edge of the specimen is characterized by river-like patterns with distinct tear ridges, which is typical of a quasi-cleavage fracture. In addition, the fracture morphology of the BM and 0.5NiCu under OCP exhibits a convergence of extended cracks in the center of the fracture. It is speculated that there are multiple crack initiation sites at the edge of the fracture, which propagate to the interior of the specimens and result in the final fracture at the center. In addition, at OCP, the fracture surface of the BM, 0.5NiCu, and LNiCu is flat, while the fracture morphology of 1NiCu still exhibits necking, demonstrating the highest SCC resistance, which is consistent with the results of SCC susceptibility in Figure 9.

**Figure 11:**
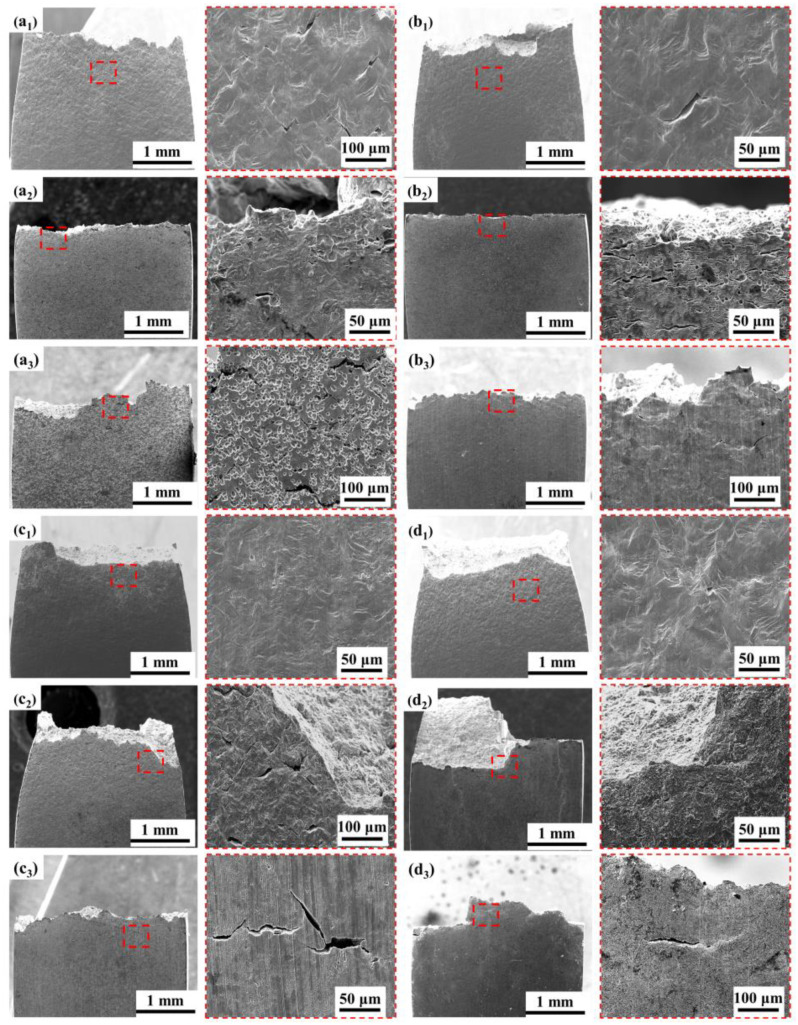
Morphology of secondary cracks of different test steels after SSRT tests in various test conditions: (**a_1_**) BM in air; (**a_2_**) BM at OCP; (**a_3_**) BM at −1200 mV; (**b_1_**) 0.5NiCu in air; (**b_2_**) 0.5NiCu at OCP; (**b_3_**) 0.5NiCu at −1200 mV; (**c_1_**) 1NiCu in air; (**c_2_**) 1NiCu at OCP; (**c_3_**) 1NiCu at −1200 mV; (**d_1_**) LNiCu in air; (**d_2_**) LNiCu at OCP; (**d_3_**) LNiCu at −1200 mV (The red tangles are key observation areas and the morphology at magnified view).

Secondary cracks of the test anchor steels were also carefully analyzed after the SSRT tests, as seen in Figure 11, which shows the side surface of the specimens. When tested in air, secondary cracks are present on the side face of the tested BM specimen but are not apparent on the microalloyed steels. When tested in solution, secondary cracks can be observed in all test specimens. At OCP, cracks with a big, open mouth can be observed, which is a sign of SCC caused by localized AD [29]. When tested at −1200 mV, the cracks are thin and shallow, which are typical of hydrogen-induced cracking (HIC) [30]. In addition, at OCP, the BM and 0.5NiCu show obvious pitting corrosion, which is less evident in the 1NiCu and LNiCu specimens as more Ni and Cu inhibit localized AD. At −1200 mV, in comparison to 1NiCu, the secondary cracks of LNiCu are longer and deeper, which indicates that the decreased C content deteriorates HE resistance. In summary, 1NiCu possesses the highest SCC resistance for inhibiting both AD and HE.

## 4. Discussion

### 4.1. Effect of Alloy Elements on the Electrochemical Mechanism

The underground mine environment is a harsh corrosive medium with multiphase flow containing CO_2_, SO_2_, and H_2_S. These gases are dissolved in water and form CO_3_^2−^ and HSO_3_^−^. Therefore, the steel surface is covered with an acidic electrolyte film, which facilitates corrosion. In the coal mine environment, H_2_S and SO_2_ dissolve in the thin liquid film and form HSO_3_^−^, which acidifies the electrolyte film [11,31]. Additionally, HSO_3_^−^ is unstable and can be easily oxidized to H_2_SO_4_, which further acidifies the electrolyte film, as shown in chemical reaction Equation (5) [32].

2HSO_3_^−^ + O_2_ → SO_4_^2−^ + 2H^+^ (5)

In consequence, anchor steels suffer from severe corrosion problems, and the electrochemical process is as follows. The anodic reaction is the dissolution of iron:

Fe + 2e^−^ → Fe^2+^ (6)

The cathodic reaction is the combination of oxygen reduction and hydrogen evolution:

O_2_ + 4e^−^ + 4H^+^ → 2H_2_O (7)

2H^+^ + 2e^−^ → H_2_ (8)

The results in this work prove that microalloying improves corrosion resistance by making a difference in the electrochemical process, which inhibits anodic dissolution and accelerates the cathodic process as illustrated in Figure 7. Compared to the BM, the microalloyed steels Sb, Nb, Ni, and Cu were added, and the content of C was reduced in 1NiCu and LNiCu. The role of the discrete alloying elements is discussed as follows.

As for the effect of Sb, based on the Pourbaix diagram [33], Sb mainly exists in the form of Sb_2_O_3_ and Sb_2_O_5_ in the rust layer [34,35]. The reaction of Sb in the corrosion process is as follows:

2Sb + 3H_2_O → Sb_2_O_3_ + 6H^+^ + 6e^−^ (9)

Sb_2_O_3_ + 2H_2_O → Sb_2_O_5_ + 4H^+^ + 4e^−^ (10)

During the process, H^+^ can be produced simultaneously, which can inhibit the anodic reactions and reduction of HSO_3_^−^. Thus, Sb inhibits anodic dissolution and enhances corrosion resistance. Additionally, Sb can interact with Cu and facilitate the formation of Cu-containing compounds in the product film, as reported in Le’s work [36], inhibiting the anodic dissolution process. Nb can form NbC nanoprecipitates, which act as hydrogen traps and improve HE resistance.

As for Cu effects, Cu inhibits anodic dissolution by promoting the formation of protective corrosion product film, but the improvement is obvious only when the addition exceeds a critical value. In addition, at the early stage of corrosion before forming the protective rust layer, Cu accelerates the corrosion process. As a result, the polarization resistance of 0.5NiCu is lower than the BM. 1NiCu exhibits a high resistance to corrosion and SCC with greater Cu and Ni contents. Ni is present in the rust layer as protective divalent products, including NiFe_2_O_4_, which has a negative electrical effect [37]. NiFe_2_O_4_ can make the inner rust layer generate cation selectivity to effectively resist corrosive ions and significantly alleviate the corrosion behavior of low-alloy, high-strength steel [38].

As for the effect of C, studies have shown that many hydrogen defects appear in the microstructure of high-strength steel after fracture in a humid environment. Thus, hydrogen entering the matrix will accumulate in special microstructures, such as carbides, reduce the interface bonding force, increase the cracking tendency of the interface, and promote the initiation and expansion of microcracks under the action of tensile stress. When the carbides grow, the large matrix–carbide boundary becomes a strong hydrogen trap, so the susceptibility to hydrogen embrittlement increases [39,40]. Figure 11 proves that the BM steel has many deep corrosion pits, possibly due to the presence of carbides, and preferential corrosion occurs around the carbides. 0.5NiCu steel without decarburization treatment shows the same problem. After the decarburization treatment of 1NiCu and LNiCu, the number of corrosion pits on the side of the fracture significantly decreases. Therefore, the decarburization treatment helps to reduce the hydrogen collection in the matrix.

### 4.2. Effect of Alloy Elements on the SCC Mechanism

**Figure 12:**
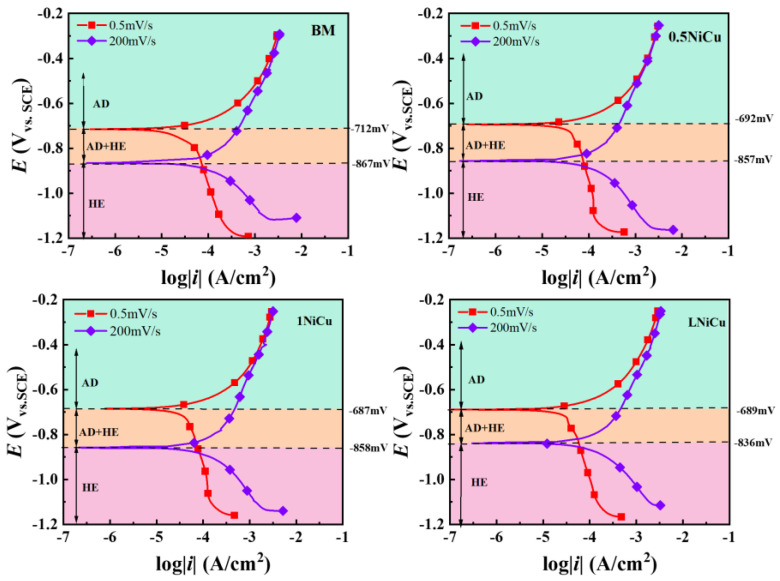
Potentiodynamic polarization curves of prepared steels at 0.5 mV/s and 200 mV/s sweep speeds.

Potentiodynamic curves at the slow scan rate of 0.5 mV/s and fast scan rate of 200 mV/s were used to clarify the effect of microalloying on electrochemical factors. The potentiodynamic polarization curves are shown in Figure 12. The comparison of the steady state and steady-state stress corrosion mechanisms identifies three regions with different corrosion mechanisms: AD, AD + HE, and HE [41,42].

Liu [42] proposed to quickly predict the potential range of each mechanism through the fast and slow sweep polarization curves and preliminarily determine the stress corrosion mechanism. According to Figure 12, the potential ranges that correspond to the stress corrosion mechanisms of the four low-alloy, high-strength steels can be determined as shown in Figure 12, and the specific data are shown in Table 5. Adding microalloying elements increases the potential range of the AD mechanism, indicating that Ni, Nb, and Sb have an inhibitory effect on anodic dissolution ability. In a similar study, Zhang [43] found that bainitic steels were more resistant to HE after adding 0.055% Nb. Both reversible and irreversible hydrogen traps are increased by Nb in steels [44,45]. It is speculated that the improvement of the hydrogen evolution corrosion potential largely depends on Nb.

With the decrease in carbon content, the anodic dissolution potential range of the 0.5NiCu and LNiCu low-alloy steels hardly changes, which indicates that the decarburization treatment does not significantly affect the anodic dissolution. With the addition of microalloying elements, the potential range of the HE mechanism slightly increases. With identical microalloying, the potential range of the HE mechanism significantly moves up in the decarburization treatment, which indicates that decarburization has a certain inhibitory effect on hydrogen embrittlement. Studies have shown that the formation of crack-sensitive paths in the process of stress corrosion may be closely related to C atoms from grain boundary defects. The presence of carbon or transition carbides at grain boundaries increases the stress corrosion sensitivity. It inhibits the dislocation movement away from the plastic deformation region associated with the crack, so it provides an adsorption site for anions on the crack surface, reduces the binding of adjacent iron atoms, and causes rapid failure.

## 5. Conclusions

Several conclusions can be drawn from the results and discussion:

(1) The four kinds of anchor steels have a similar microstructure, which is mainly composed of pearlite.
(2) The combined addition Ni, Cu, and Sb can improve the mechanical properties of the anchor steel, while reducing C content decreases tensile strength as a result of the loss of the solution-strengthening effect.
(3) The addition of Sb, Cu, Ni, and reducing the content of C enhances the resistance to corrosion and SCC by mitigating AD, while adding Nb improves SCC by inhibiting HE. The combined action of 1% Ni, 0.5% Cu, 0.05% Nb, 0.1% Sb, and 0.5% C presents the highest SCC resistance.

## Figures and Tables

**Table 1:** The chemical compositions (wt.%) of the tested anchor steels.

	C	Si	Mn	Cr	Ni	Cu	Nb	Sb	P	S
BM	0.71	0.21	0.80	0.20	0	0	0	0	0.018	0.002
0.5NiCu	0.71	0.21	0.80	0.20	0.49	0.51	0.046	0.11	0.021	0.016
1NiCu	0.53	0.21	0.80	0.20	0.98	0.51	0.046	0.11	0.022	0.012
LNiCu	0.34	0.21	0.80	0.20	0.98	0.51	0.046	0.11	0.015	0.014

**Table 2:** The chemical composition of the simulated mine environment (g/L).

NaCl	KNO_3_	Na_2_SO_4_	NaHCO_3_	NaHSO_3_
0.25	0.1	0.50	1.00	1.00

**Table 3:** The mechanical properties of the test steels.

Steels	Tensile Strength (MPa)	Yield Strength (MPa)	Elongation Rate (%)	Reduction in Area (%)
BM	962 ± 85	525 ± 57	9.14 ± 1.5	31.97 ± 2.9
0.5NiCu	1046 ± 99	604 ± 78	8.65 ± 1.2	32.73 ± 3.8
1NiCu	907 ± 83	557 ± 55	9.30 ± 2.0	45.65 ± 5.5
LNiCu	856 ± 89	551 ± 65	7.60 ± 1.5	37.01 ± 3.5

**Table 4:** Fitting results of the EIS curves of all test steels.

Steel	*R_s_*/Ω·cm^2^	*Q_f_* × 10^−4^/Ω^−1^cm^−2^s^n^	*n_f_*	*R_pore_*/Ω·cm^2^	*Q_dl_* × 10^−4^/Ω^−1^cm^−2^s^n^	*n_dl_*	*R_ct_*/Ω·cm^2^	*χ*^2^ × 10^−4^
BM	105.5	3.99	0.93	57.37	5.94	0.95	239.8	3.64
0.5NiCu	103.4	7.55	0.82	32.23	10.43	0.83	247.8	1.53
1NiCu	107.1	4.77	0.86	49.25	7.37	0.90	281.1	2.05
LNiCu	111.7	6.93	0.82	27.22	11.9	0.82	195.5	1.12

**Table 5:** Potential range corresponding to three stress corrosion mechanisms of the anchor steels (V vs. SCE).

Steels	AD	AD + HE	HE
BM	>−0.712 V	−0.712~−0.867 V	<−0.867 V
0.5NiCu	>−0.692 V	−0.857~−0.692 V	<−0.857 V
1NiCu	>−0.687 V	−0.858~−0.687 V	<−0.858 V
LNiCu	>−0.689 V	−0.836~−0.688 V	<−0.836 V

## Data Availability

The raw/processed data required to reproduce these findings cannot be shared at this time as the data is related to an ongoing study.
